# Differences in clinical features of cluster headache between drinkers and nondrinkers in Japan

**DOI:** 10.1371/journal.pone.0224407

**Published:** 2019-11-20

**Authors:** Noboru Imai, Eiji Kitamura

**Affiliations:** 1 Department of Neurology, Japanese Red Cross Shizuoka Hospital, Shizuoka, Shizuoka, Japan; 2 Department of Neurology, Kitasato University, Sagamihara, Kanagawa, Japan; Taipei Veterans General Hospital, TAIWAN

## Abstract

**Objective:**

Alcohol has been recognized as the main trigger for a cluster headache attack, but clinical features to distinguish between cluster headache in drinkers and nondrinkers are unclear. Thus, the present study aimed to investigate the differences in clinical features of cluster headache between drinkers and nondrinkers.

**Methods:**

This retrospective, observational study compared the clinical features of cluster headache between drinkers and nondrinkers among patients who were diagnosed with cluster headache between November 2004 and April 2018 at the Japanese Red Cross Shizuoka Hospital. Demographic and clinical data were collected from medical records and/or by patient interview.

**Results:**

Of 131 patients, 98 (75%) were drinkers, and 33 (25%) were nondrinkers. Compared with nondrinkers, drinkers had significantly more frequent conjunctival injection (43% vs. 21%, *p* = 0.037) but significantly less frequent nasal congestion (31% vs. 52%, *p* = 0.0037), vomiting (11% vs. 30%, *p* = 0.014), and photophobia (29% vs. 45%, *p* = 0.008).

**Conclusion:**

Among individuals with cluster headache, the frequencies of conjunctival injection, nasal congestion, vomiting, and photophobia were different between drinkers and nondrinkers. These results suggested that drinking might influence the responses of the cranial autonomic reflex with respect to conjunctival injection or nasal congestion.

## 1. Introduction

Cluster headache (CH) is a primary headache syndrome that is characterized by recurrent brief attacks of severe, exclusively unilateral pain that lasts for 15–180 min and has been associated with cranial autonomic symptoms and restlessness [1]. Alcohol is widely recognized as the main trigger for CH [1]. However, most previous studies on alcohol-triggered CH were confined to Caucasian populations [2–9], and only two reports were from Asia, specifically Taiwan and China [10, 11]. These studies described the frequency at which an attack could be elicited by drinking. However, only a few studies described the differences in clinical features of CH between drinkers and nondrinkers.

Our previous study showed ethnic differences in the prevalence of chronic CH and in the uncoupling of the sense of restlessness and restless behavior between Japanese and Taiwanese patients and Caucasian patients [12]. Nevertheless, there is limited information regarding the clinical features of CH according to alcohol drinking patterns. The present study aimed to clarify the alcohol-related clinical features of CH patients in Japan and to investigate differences in these clinical features between drinkers and nondrinkers.

## 2. Materials and methods

### 2.1 Patients

The study population comprised patients who were diagnosed with CH using the criteria of the International Classification of Headache Disorders 3 beta version [1] during first consultation or follow-up between November 2004 and April 2018 at the Japanese Red Cross Shizuoka Hospital. All patients underwent magnetic resonance imaging to rule out the presence of neurologic lesions.

### 2.2 Methods

Data pertaining to the following variables were collected from the medical records and/or by patient interview: age; age at onset; sex; body mass index; type of CH; laterality and sites of headache; pain intensity; autonomic features; additional symptoms of nausea, vomiting, photophobia, phonophobia, and feeling of restlessness/restless behavior during attacks; frequency of the attacks; duration of the attacks; time of onset of the attacks; history of alcohol intake; history of new attacks triggered by drinking; and abstinence from alcoholic drinks during the CH period. Pain intensity was estimated using a visual analog scale. Patients were classified as drinkers or nondrinkers according to the history of alcohol intake. Nondrinkers were defined as those who had abstained from alcoholic beverages for more than 1 year, and all remaining patients were defined as drinkers and were further classified as habitual drinkers or social drinkers. Habitual drinkers were defined as those who drank approximately 180 mL of alcohol per day at least 3 days per week, in accordance with the definition of the Japanese Ministry of Health, Labour and Welfare [13], and all remaining drinkers were defined as social drinkers.

### 2.3 Standard protocol approval and patient consent

This study protocol was approved by the ethics committee of the Japanese Red Cross Shizuoka Hospital (approval number 2018–08) and conformed to the ethical standards specified in the Declaration of Helsinki. All patients received written and verbal information about the study and provided written informed consent prior to inclusion.

### 2.4 Statistical analysis

Data are presented as means ± standard deviations. Student’s *t*-test was used to compare continuous variables, whereas the chi-squared test was used to compare categorical variables. Fisher’s exact test was used to compare variables with low frequency (i.e., <5 observations). All tests were two-tailed, and we considered an alpha level of <0.05 to be statistically significant. SPSS Statistics software (version 20.0, IBM Corp., Armonk, NY, USA) was used for all statistical analyses.

## 3. Results

### 3.1 Participants

A total of 157 patients with CH were enrolled. Twenty-six patients were excluded from the study because of missing data regarding alcohol-related clinical features. Finally, 131 patients with CH (mean age, 37.9 ± 10.3 years; 76% men) were evaluated in this study. No significant differences in age, sex, or CH subtype were observed between evaluated and excluded patients.

### 3.2 Comparison of demographic and clinical features between drinkers and nondrinkers

Of the 131 patients, 98 (75%) were drinkers, and 33 (25%) were nondrinkers. Compared with the nondrinkers, a significantly greater proportion of drinkers were men, and the drinkers had a significantly higher mean body mass index (Table 1). Conjunctival injection was significantly more common among drinkers than among nondrinkers, whereas nasal congestion, vomiting, and photophobia were significantly less common among drinkers than among nondrinkers (Table 2). No significant differences were observed between the drinkers and nondrinkers with regard to the following factors: age at first consultation; age at onset; type of CH; current smoking status; laterality of headache; visual analog scale score; duration, frequency, and time of onset of the attacks; and bout frequency (Tables 1–4).

**Table 1:** Demographic and clinical variables of patients with cluster headache.

Patient characteristics	Drinkers (*n* = 98, 75%)	Nondrinkers (*n* = 33, 25%)	*p*
Age (years) (mean ± SD)	37.9 ± 9.7	37.7 ± 12.0	0.927
Age at onset (years) (mean ± SD)	30.1 ± 11.1	29.6 ± 13.9	0.826
Sex, male, n (%)	81 (83)	18 (55)	**0.001**
Body mass index (mean ± SD)	22.9 ± 2.	21.3 ± 3.3	**0.011**
Chronic type of cluster headache, n (%)	3 (3)	1 (3)	0.993
Current smoking, n (%)	47 (48)	13 (39)	0.391

SD, standard deviation

**Table 2:** Cranial autonomic and additional features in drinkers and nondrinkers.

Features	Drinkers	Nondrinkers	*p*
Autonomic			
Lacrimation	70 (71)	23 (70)	0.828
Conjunctival injection	42 (43)	7 (21)	**0.037**
Rhinorrhea	55 (56)	20 (61)	0.689
Nasal congestion	30 (31)	17 (52)	**0.037**
Eyelid edema	5 (5)	4 (12)	0.228
Facial sweating	22 (2%)	7 (21)	1.000
Ptosis	11 (11)	4 (12)	1.000
Additional			1.000
Nausea	45 (46)	17 (52)	0.688
Vomiting	11 (11)	10 (30)	**0.014**
Photophobia	28 (29)	15 (45)	**0.008**
Phonophobia	23 (23)	13 (39)	0.113
Visual aura	2 (2)	1 (3)	1.000
Sense of restlessness	55 (56)	17 (52)	0.689
Pacing	31 (32)	11 (33)	0.833
Aggravation by physical activities	18 (18)	8 (24)	0.459

Data are shown as n (%).

**Table 3:** Headache characteristics in drinkers and nondrinkers.

Headache characteristics	Drinkers	Nondrinkers	*p*
Location, n (%)			
Retro-orbital	79 (81%)	27 (82%)	0.843
Temporal	58 (59%)	20 (61%)	0.886
Forehead	27 (28%)	11 (33%)	0.527
Occipital	19 (19%)	7 (21%)	0.82
Upper teeth	14 (14%)	5 (15%)	0.903
Vertex	11 (11%)	5 (15%)	0.587
Cheek	9 (9%)	7 (21%)	0.068
Nose	5 (5%)	2 (6%)	0.832
Jaw	6 (6%)	0 (0%)	0.146
Neck	5 (5%)	1 (3%)	0.622
Ear	4 (4%)	1 (3%)	0.785
Shoulder	2 (2%)	0 (0%)	0.408
Laterality, n (%)			0.373
Right-sided attacks only	45 (46%)	20 (61%)	
Left-sided attacks only	41 (42%)	10 (30%)	
Side changes during bouts	2 (2%)	0 (0%)	
Side changes between bouts	6 (6%)	3 (9%)	
Side changes during and between bouts	4 (4%)	0 (0%)	
Visual analog scale score (mean ± SD)	93.4 ± 11.0	91.3 ± 15.3	0.392

SD, standard deviation

**Table 4:** Attack characteristics.

Attack characteristics	Drinkers	Nondrinkers	*p*
Frequency			0.781
Less than 1 time/day	19 (19%)	5 (15%)	
From 1 time to less than 2 times/day	62 (63%)	20 (61%)	
From 2 times to less than 3 times/day	7 (7%)	4 (12%)	
More than 3 times/day	10 (10%)	4 (12%)	
Duration			0.435
Less than 1 h	22 (22%)	7 (21%)	
From 1 h to less than 2 h	49 (50%)	14 (42%)	
From 2 h to less than 3 h	17 (17%)	9 (27%)	
More than 3 h	8 (8%)	1 (3%)	
Varies with each attack	2 (2%)	2 (6%)	
Time of onset			0.638
Daytime only	7 (7%)	3 (9%)	
Equally nocturnal and daytime	22 (22%)	3 (9%)	
Mostly daytime	21 (22%)	7 (21%)	
Mostly nocturnal	31 (32%)	14 (42%)	
Nocturnal only	13 (13%)	5 (15%)	
Varies among cluster periods	4 (4%)	1 (3%)	
Bout frequency, n (%)			
Less than 1 time/year	39 (40%)	18 (55%)	
1 time/year	28 (29%)	7 (21%)	
From 1 to 2 times/year	14 (14%)	5 (15%)	
More than 2 times/year	4 (4%)	1 (3%)	
Single bout	10 (10%)	1 (3%)	
Chronic	3 (3%)	1 (3%)	

Data are shown as n (%).

### 3.3 Alcohol-related clinical features during the cluster headache period

Of the 98 drinkers, 78 (60%) were habitual drinkers, and 20 (15%) were social drinkers. All habitual drinkers reported drinking during the CH period, and no social drinker consumed alcohol during the CH period. Alcohol intake triggered a new attack in 74 patients who were habitual drinkers (95% of habitual drinkers), but not in four patients who were habitual drinkers (5% of habitual drinkers).

Compared with social drinkers, habitual drinkers were significantly older at first consultation (Table 5). No significant differences were observed between habitual drinkers and social drinkers with regard to the following factors: age at onset; type of CH; current smoking status; laterality of headache; visual analog scale score; autonomic features; additional features; duration, frequency, and time of onset of the attacks; and bout frequency (S1–S3 Tables).

**Table 5:** Demographic and clinical variables in habitual drinkers and social drinkers.

Patient characteristics	Habitual drinkers (*n* = 78)	Social drinkers (*n* = 20)	*p*
Age (years) (mean ± SD)	39.1 ± 9.7	33.3 ± 8.2	**0.016**
Age at onset (years) (mean ± SD)	30.3 ± 11.3	29.3 ± 10.7	0.73
Sex, male, n (%)	66 (78)	15 (75)	0.33
Body mass index (mean ± SD)	22.7 ± 2.9	23.4 ± 3.2	0.370
Chronic type of cluster headache, n (%)	1 (1)	2 (10)	0.105
Current smoking, n (%)	41 (53)	10 (50)	1.000
Visual analog scale score (mean ± SD)	94.1 ± 9.8	90.7 ± 15.0	0.224

SD, standard deviation

Of the 78 habitual drinkers, 72 (92%) stopped drinking alcohol during the CH period. Among the four patients in whom a new attack was not provoked by drinking, none stopped drinking. Two patients continued drinking despite the occurrence of an attack during the CH period.

## 4. Discussion

Our study showed that 75% of CH patients were drinkers. Moreover, compared with the nondrinkers, a significantly greater proportion of men were drinkers, and these drinkers had significantly more frequent conjunctival injection and less frequent nasal congestion, vomiting, and photophobia.

In this study, the proportion of Japanese patients with CH who were drinkers (75%) was similar to the reported proportion of patients who consumed alcohol in Sweden (72%) [8], higher than the proportions of patients reported in the United States (65%) and Denmark (61%) [7, 9], and lower than the proportions of patients reported in the United Kingdom, Germany, and Italy (83%–84%) [3, 4,14]. Our study also showed that conjunctival injection was significantly more common among drinkers than among nondrinkers, whereas nasal congestion, vomiting, and photophobia were significantly less common. Conjunctival injection and nasal congestion are parasympathetic symptoms of CH that are presumed to be caused by either a direct effect of the hypothalamus or a reflex activation of the parasympathetic outflow from the superior salivatory nucleus, predominantly through the pterygopalatine ganglion [15]. Although conjunctival injection and nasal congestion are thought to be caused by the same mechanism, our study revealed different proportions of CH patients with conjunctival injection and nasal congestion between drinkers and nondrinkers. We speculate that drinking alcoholic beverages may alter the differences in the responses of the cranial autonomic reflex between conjunctival injection and nasal congestion. Nausea, vomiting, photophobia, and phonophobia are frequently observed in patients with CH and have been described as “migraine-like accompanying features” (MLF) [16]. Taga et al. reported that there were more women among individuals who had CH with MLF compared with individuals who had CH without MLF. Furthermore, individuals who had CH with MLF were younger at the time of CH onset, had longer duration of CH, and had more frequent ptosis, sweating, miosis, and osmophobia, but had similar daily alcohol intake [16]. The differences between the results of our study and those of the previous study may have been a result of different study designs and/or social and ethnic differences.

Alcohol intake triggered a new CH attack in 22% to 69% of patients in Western countries [2–9], in 24% of patients in Taiwan [10], in 56% of patients in China [10], and in 57% of patients in our study. Levi et al. reported that, among Swedish patients, alcohol elicited a headache attack in 79%, but reduced a current episode of headache in 9% [2]. In a study conducted by Rozen et al. in the United States, 65% of the surveyed patients consumed alcohol, and 52% experienced a CH attack after drinking alcohol [7]. In 80% of the surveyed patients who consumed alcohol, a headache attack had been triggered by drinking. In our study, 95% of patients with a history of drinking during the CH period had experienced a headache attack after drinking. The higher frequency of drinking-provoked headache attack among current drinkers in Japan than in Western countries may reflect ethnic differences.

Our study showed that 92% of habitual drinkers stopped drinking alcohol during the CH period. Among drinkers in the United States, the frequency of abstinence from alcohol during a CH period was 85% [7], and Levi et al. reported that alcohol consumption decreased in 79% of CH patients in Sweden [2]. In Denmark, Lund et al. revealed that 14.3% of patients continued drinking alcohol during attack periods [9], and in France, Donnet et al. demonstrated that 42% of patients with chronic CH were regular alcohol consumers [5]. The relatively high frequency of alcohol abstinence during CH periods in Japan may also reflect ethnic differences.

### 4.1 Study limitations

This study had some limitations. First, the data were collected retrospectively. Second, the study was performed at a single center with a small sample of patients; these were key limitations in this study. Third, our evaluation of the trigger factor for headache was based on patient recall.

## 5. Conclusions

This study of patients with CH showed that conjunctival injection was significantly more common among drinkers than among nondrinkers, whereas nasal congestion, vomiting, and photophobia were significantly less common. Drinking may result in cranial autonomic reflex responses that differentially affect conjunctival injection and nasal congestion.

## Supporting information

S1 Table Headache characteristics in habitual drinkers and social drinkers. (DOCX)

S2 Table Cranial autonomic and additional features in habitual drinkers and social drinkers. (DOCX)

S3 Table Attack characteristics in drinkers and social drinkers. (DOCX)
