# Supplementary material for: Differences in clinical features of cluster headache between drinkers and nondrinkers in Japan
Source: PLoS One. 2019 Nov 20;14(11):e0224407. doi: 10.1371/journal.pone.0224407 (PMC6867697; doi:10.1371/journal.pone.0224407)
Supplement: S1 Table — (DOCX) [file pone.0224407.s001.docx]

S1 Table. Headache characteristics in habitual drinkers and social drinkers

| Headache characteristics | Habitual drinkers | Social drinkers | *p* |
| --- | --- | --- | --- |
| Location, n (%)  Retro-Orbital  Temporal  Forehead  Occipital  Upper Teeth  Vertex  Cheek  Nose  Jaw  Neck  Ear  Shoulder | 64 (82%) 47 (60%) 19 (24%) 17 (22%) 14 (18%) 10 (13%) 6 (8%) 5 5%) 6 (8%) 5 (6%) 4 (4%) 2 (3%) | 16 (80%) 11 (55%) 8 (40%) 2 (10%) 0 (0%) 2 (10%) 3 (15%) 0 (0%) 0 (0%) 0 (0%) 0 (0%) 0 (0%) | 0.758 0.800 0.172 0.346 0.067 1.000 0.383 0.580 0.341 0.580 0.579 1.000 |
| Laterality, n (%)   Right-sided attacks only  Left-sided attacks only  Side changes within the bouts  Side changes between bouts | 37 (45%) 35 (45%) 1 (1%) 5 (6%) | 10 (50%) 8 (40%) 1 5%) 1 (5%) | 1.000 |
