# Supplementary material for: Differences in clinical features of cluster headache between drinkers and nondrinkers in Japan
Source: PLoS One. 2019 Nov 20;14(11):e0224407. doi: 10.1371/journal.pone.0224407 (PMC6867697; doi:10.1371/journal.pone.0224407)
Supplement: S2 Table — (DOCX) [file pone.0224407.s002.docx]

S2 Table. Cranial autonomic and additional features in habitual drinkers and social drinkers

| Features | Habitual drinkers | Social drinkers | *p* |
| --- | --- | --- | --- |
| Autonomic  Lacrimation  Conjunctival injection Rhinorrhea Nasal congestion  Eyelid edema  Facial sweating  Ptosis | 58 (74)  37 (47)  44 (56)  25 (32)  3 (4)  14 (18%)  7 (9) | 12 (60)  5 (25)  11 (55)  5 (25)  2 (10)  8 (40)  4 (20) | 0.267  0.082  1.000  0.600  0.269  0.067  0.228 |
| Additional  Nausea Vomiting Photophobia  Phonophobia  Visual aura  Sense of restlessness  Pacing  Aggravation by physical activities | 38 (49)  10 (13)  25 (32)  19 (24)  3 (3)  46 (59)  25 (32)  13 (17) | 7 (35)  1 (5)  3 (15)  4 (20)  0 (0)  9 (45)  6 (30)  5 (25) | 0.321  0.452  0.171  0.776  1.000  0.316  1.000  0.5.17 |

Data are shown as n (%).
