# Supplementary material for: Differences in clinical features of cluster headache between drinkers and nondrinkers in Japan
Source: PLoS One. 2019 Nov 20;14(11):e0224407. doi: 10.1371/journal.pone.0224407 (PMC6867697; doi:10.1371/journal.pone.0224407)
Supplement: S3 Table — (DOCX) [file pone.0224407.s003.docx]

S3 Table. Attack characteristics in drinkers and social drinkers

| Patient characteristics | Habitual drinkers | Social drinkers | *p* |
| --- | --- | --- | --- |
| Frequency  Less than 1 time / day  From 1 time to less than 2 times / day  From 2 times to less than 3 times /day  More than 3 times / day | 16 (21%) 46 (59%) 7 (9%) 9 (12%) | 3 (15%) 16 (80%) 0 (0%) 1 (5%) | 0.281 |
| Duration  Less than 1h  From 1 h to less than 2 h  From 2 h to less than 3 h  More than 3 h  Varies with each attack | 17 (22%) 38 (49%) 14 (18%) 7 (9%) 2 (3%) | 5 (25%) 11 (55%) 3 (15%) 1 (5%) 0 (0%) | 0.894 |
| Time of onset  Daytime only  Equally nocturnal and daytime  Mostly daytime  Mostly nocturnal  Nocturnal only  Varies among cluster periods | 5 (6%) 18 (23%) 13 (17%) 26 (33%) 12 (15%) 4 (5%) | 2 (10%) 4 (20%) 87 (40%) 5 (25%) 1 (5%) 0 (0%) | 0.216 |
| Bout frequency, n (%)  Less than 1 time / year  1 time / year  From 1 to 2 times / year  More than 2 times / year   Single bout  Chronic | 39 (40%) 28 (29%) 14 (14%) 4 (4%) 10 (10%) 3 (3%) | 18 (55%) 7 (21%) 5 (15%) 1 (3%) 1 (3%) 1 (3%) |  |

Data are shown as n (%).
